# Cardiovascular magnetic resonance assessment of ventricular function and myocardial scarring before and early after repair of anomalous left coronary artery from the pulmonary artery

**DOI:** 10.1186/1532-429X-16-3

**Published:** 2014-01-05

**Authors:** Heiner Latus, Kerstin Gummel, Stefan Rupp, Matthias Mueller, Christian Jux, Gunter Kerst, Hakan Akintuerk, Juergen Bauer, Dietmar Schranz, Christian Apitz

**Affiliations:** 1 Pediatric Heart Center, University Children’s Hospital, Giessen, Germany; 2 Department of Pediatric Cardiology, University Children’s Hospital Münster, Münster, Germany; 3 Division of Pediatric Cardiovascular Surgery, University Children’s Hospital, Giessen, Germany; 4 Pediatric Heart Center, Justus-Liebig-University of Giessen, Feulgenstr. 12, D-35392 Giessen, Germany

**Keywords:** ALCAPA syndrome, Left ventricular remodelling, Cardiovascular magnetic resonance, Hibernation, Infarction

## Abstract

**Background:**

In patients with anomalous left coronary artery from the pulmonary artery (ALCAPA) left ventricular (LV) dilatation and dysfunction evolves due to diminished myocardial perfusion caused by coronary steal phenomenon. Using late gadolinium enhanced cardiovascular magnetic resonance (LGE-CMR) imaging, myocardial scarring has been shown in ALCAPA patients late after repair, however the incidence of scarring before surgery and its impact on postoperative course after surgical repair remained unknown.

**Methods:**

8 ALCAPA-patients (mean age 10.0 ± 5.8 months) underwent CMR before and early after (mean 4.9 ± 2.5 months) coronary reimplantation procedures. CMR included functional analysis and LGE for detection of myocardial scars.

**Results:**

LV dilatation (mean LVEDVI 171 ± 94 ml/m^2^) and dysfunction (mean LV-EF 22 ± 10 %) was present in all patients and improved significantly after surgery (mean LVEDV 68 ± 42 ml/m^2^, p = 0.02; mean LV-EF 58 ± 19 %, p < 0.001). Preoperative CMR revealed myocardial scarring in 2 of the 8 patients and did not predict postoperative course. At follow-up CMR, one LGE-positive patient showed delayed recovery of LV function while myocardial scarring was still present in both patients. In two patients new-onset transmural scarring was found, although functional recovery after operation was sufficient. One of them showed a stenosis of the left coronary artery and required resurgery.

**Conclusions:**

Despite diminished myocardial perfusion and severely compromised LV function, myocardial scarring was preoperatively only infrequently present. Improvement of myocardial function was independent of new-onset scarring while the impact of preoperative scarring still needs to be defined.

## Background

Anomalous origin of the left coronary artery from the pulmonary artery (ALCAPA) is a rare congenital cardiovascular anomaly that occurs in approximately 1 of 300, 000 live births [1,2]. Ischemic left ventricular (LV) dilatation and dysfunction typically evolves in patients older than three months of age due to diminished myocardial perfusion caused by coronary steal phenomenon. Immediate corrective surgery using coronary reimplantation procedures establishes a two-coronary system circulation that leads to quick recovery of myocardial function in the majority of patients within the first postoperative year resulting in excellent overall survival [3-7].

Although there is evidence that the condition produces chronic myocardial ischemia and induces infarction prior to corrective surgery [8-10], the presence of myocardial scarring in ALCAPA-patients at an early age has not been studied so far. The knowledge about the existence of myocardial scarring prior to surgery is inasmuch relevant as Shivalkar and colleagues have hypothesized the classic concept of a hibernating myocardium in ALCAPA-syndrome in which chronic hypoperfusion results, in part, in viable but dysfunctional myocardium with a variable degree of fibrosis. This unique condition, which is characterized by severely depressed cardiac function with ischemic but yet viable myocardium, in turn, has been accounted for the dramatic improvement in LV function after successful coronary reimplantation.

Considering the complexity of assessing hibernating myocardium, different imaging modalities provide different surrogate definitions of hibernation [11,12]. Late gadolinium enhancement (LGE) is an established cardiovascular magnetic resonance (CMR) technique that allows reliable detection of myocardial scarring including exact scar localization and determination of size of necrosis/fibrosis. Thereby, LGE-CMR has the potential to identify abnormal hibernating myocardium, defined as absence of scar in areas with hypokinesia at rest, and distinguishing it from irreversibly damaged necrotic tissue [13].

Accordingly, the aim of the study was to evaluate the frequency and patterns of myocardial scarring in ALCAPA-patients before surgical repair using contrast enhanced cardiovascular magnetic resonance (CE-CMR) and to evaluate its impact on postoperative course. A short-term follow-up CMR after coronary reimplantation procedure was conducted to quantify ventricular recovery and to re-assess myocardial viability.

## Methods

### Study population

The study enrolled eight patients (four females) who had diagnosis of ALCAPA between March 2009 and April 2012 (Table 1). Mean age at diagnosis was 5.1 ± 4.4 months (mean weight 5.6 ± 2.2 kg). Anomalous origin of the left coronary artery was diagnosed preoperatively by cardiac catheterization in seven patients and by echocardiography in one patient. Associated cardiac defects included a small ASD II and a perimembraneous ventricular septal defect (VSD) in one patient, respectively. All patients had an initial CMR examination with assessment of ventricular dimensions and function including myocardial delayed enhancement. Subsequently, patients underwent prompt coronary reimplantation procedures. A second CMR study was conducted early after repair (mean time interval 4.9 ± 2.5 months between initial and follow-up CMR) to evaluate functional recovery including repeated myocardial viability study to assess myocardial scarring.

**Table 1:** Demographic and clinical data of the study population

**Variable**	**Value**
**Patients, n**	8
**Male/female, n**	4/4
**Age at CMR pre-op, months**	5.1 ± 4.4
**Weight, kg**	5.6 ± 2.2
**Height, cm**	56 ± 14
**BSA, m**^**2**^	0.29 ± 0.08
**Age at CMR post-op, months**	10.0 ± 5.8
**Weight, kg**	7.9 ± 2.4
**Height, cm**	69 ± 8
**BSA, m**^**2**^	0.39 ± 0.08
**Time between CMR studies, months**	4.9 ± 2.5
**Type of repair**	
**Direct reimplantation, n**	5
**Takeuchi repair, n**	2
**CPB time, min**	124 ± 23
**ACC time, min**	69 ± 9
**Mechanical ventilation time, h**	139 ± 174
**Inotropic support, days**	13.4 ± 10.2
**ICU stay, days**	14.0 ± 9.9
**MCS, n (days)**	2 (8.5 ± 4.9)

*CMR*, cardiovascular magnetic resonance; *BSA*, body surface area; *CPB*, cardiopulmonary bypass; *ACC*, aortic cross-clamp; *ICU*, intensive care unit; *MCS*, mechanical circulatory support. Data expressed as mean and 1 standard.

Clinical data were retrospectively obtained from hospital medical records including date of birth, gender, anatomic diagnoses, type of surgical coronary reimplantation technique and data on intraoperative and early postoperative course as aortic cross clamp time, cardiopulmonary bypass time, duration of inotropic support, duration of mechanical ventilation, length of stay on intensive care unit and need for extracorporeal membrane oxygenation. Findings of the CMR studies were extracted from the routine clinical reports. Laboratory findings of B-type natriuretic peptide (BNP) and troponin I (TNI) at initial admission and at the follow-up MRI were also assessed.

The study protocol was approved by the local ethics committee (University Medical Center Giessen review board) and consent for use of anonymised CMR and clinical data for research purposes was obtained from all patients or parents of the patients.

### Cardiovascular magnetic resonance

CMR was performed on a 3-T system (Verio, Siemens, Erlangen, Germany). Images were acquired with two sixteen-elements phased array coils. Sedation with midazolam and propofol was applied in all patients but none of the patients received mechanical ventilation.

#### ***Cine CMR***

The CMR protocol included a stack of short-axis slices from the base of the heart to the apex using gradient echo (GE) sequences in free-breathing technique. The sequence parameters were: TR 56 ms, TE 2.54 ms, flip angle 12°, slice thickness 5 mm, in plane image resolution 1.4 mm × 1.4 mm × 5.0 mm. End-diastolic (maximal) and end-systolic (minimal) volumes, stroke volumes (SV) and ejection fractions (EF) for the RV and LV were calculated by dedicated software (ARGUS, Siemens, Erlangen, Germany) after the RV and LV endocardial borders were traced manually at end-systole and end-diastole. The LV mass was calculated by subtracting endocardial from epicardial volume at end-diastole and multiplying by 1.05 g/cm^3^. All parameters were adjusted to body surface area (BSA).

#### ***Late gadolinium enhanced CMR***

Gadolinium-based contrast agents are required for numerous CMR indications. Although none of the commonly used agents has been approved by the United States Food and Drug Administration (FDA) for the use in children <2 years of age, pediatric cardiologists and radiologists frequently practice contrast-based CMR even in infants and neonates [14,15]. Therefore, it remains the clinicians’ decision to thoroughly weigh benefits against risks before using gadolinium in very young children. Considering the important role of gadolinium-based contrast agents in adults with ischemic cardiomyopathy and regarding the recently reported use of LGE-CMR in two infants with ALCAPA [16], the potential benefits of assessing myocardial scarring may justify its application in these patients.

Before administration of gadolinium, renal function parameters, i.e. glomerular filtration rate, as well as creatinine levels were assessed to exclude renal dysfunction. Finally, the parents of the patients were informed about the use of gadolinium and gave consent for the contrast-based CMR scan.

LGE was assessed after intravenous injection of gadopentetate dimeglumine (Magnevist, Bayer, Leverkusen, Germany) at a dose of 0.15 mmol/kg of body weight in free-breathing technique by using a two-dimensional phase-sensitive inversion-recovery (PSIR) segmented gradient echo MR sequence in the cardiac short and long-axis planes (slice thickness 5 mm, field of view 300 mm). The inversion time was adjusted for optimal suppression of signal from normal myocardium and the images were obtained within 5–10 minutes after injection (inversion time approximately 250–350 ms). TR was 414 ms and TE 1.65 ms with a flip angle of 20°, in-plane resolution was 1.2 × 1.2 mm, slice thickness 5 mm and 3–4 signal averages. All LGE images were interpreted accordingly to American Heart Association 17-segment model. Extent of LGE was assessed using certified software (cmr^42^, Circle Cardiovascular Imaging Inc., Calgary, Canada).

### Statistical analysis

Continuous variables are presented as mean with standard deviation. Comparisons of data before and after coronary reimplantation procedures were performed using the *t*-test for paired data. Analysis was performed using GraphPad statistical software package (San Diego, California, USA). A *p* value ≤ 0.05 was considered statistically significant.

## Results

**Figure 1:**
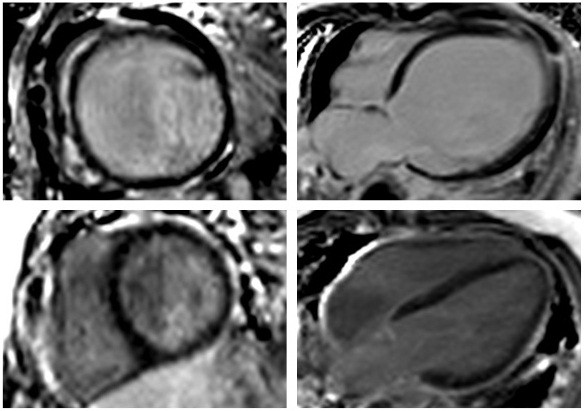
**CMR-LGE short-axis (*left*) and 4-chamber (*right*) view of a patient before (*top*) and early after (*bottom*) ALCAPA-repair.** Despite severe LV dilation and poor LV function (LVEF 11%) preoperatively, myocardial scarring was absent. Post repair, LV volume decreased and LV function recovered (LVEF 71%) while still no myocardial scar was detectable.

**Figure 2:**
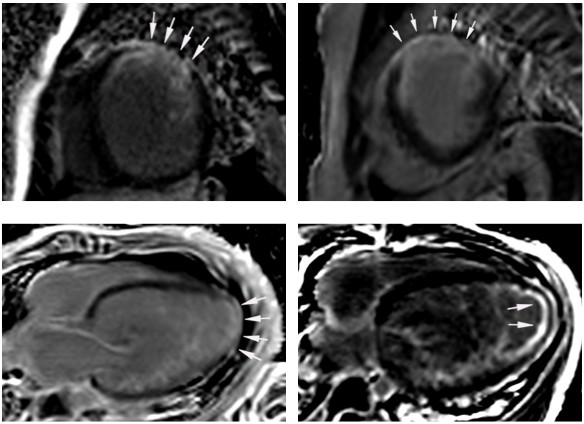
**CMR-LGE images in short-axis view of a patient who presented with transmural myocardial scarring (*arrows*) pre-repair (*top left*).** Myocardial scar increased post-repair from 9.3 to 11.4% of myocardial mass *(top right)* while functional recovery was adequate. In another patient transmural LGE in thin apical LV myocardium (4-chamber view) was detected pre-repair (*bottom left*) and was characterized as subendocardial post-repair (*bottom right*). This patient showed almost no recovery of LV dimension and function at early follow-up CMR.

Preoperative CMR showed marked LV dilatation with a mean indexed enddiastolic volume (EDV) of 171 ± 94 ml/m^2^ and a globally hypokinetic LV myocardium with impaired function (mean LV ejection fraction (EF) 22 ± 10%) (Table 2). Myocardial scarring assessed by LGE was found in 2 of the 8 patients (25%) (Figure 1) and was characterised as transmural in thin apical LV myocardium in one patient while the other LGE-positive patient showed transmural scarring at the basal and midventricular level in the antero- and inferolateral segments (Figure 2). Right ventricular function was preserved with a mean EF of 59 ± 11%.

**Table 2:** Laboratory and CMR findings before and in the short-term follow-up after ALCAPA repair

**Variable**	**Pre-repair**	**Post-repair**	**Sign. P**
**BNP, pg/mL (n = 7)**	1349 ± 765	90 ± 66	<0.001
**TNI, μg/L (n = 7)**	0.34 ± 0.26	0.02 ± 0.01	0.02
**Heart rate, /min**	126 ± 10	122 ± 16	0.37
**LVEDVi, mL/m**^**2**^	171 ± 94	68 ± 42	0.02
**LVESVi, mL/m**^**2**^	139 ± 95	35 ± 45	0.02
**LVSVi, mL/m**^**2**^	32 ± 11	34 ± 9	0.49
**LVEF, %**	22 ± 10	58 ± 19	<0.001
**MM, g/m**^**2**^	91 ± 48	61 ± 30	0.04
**LGE +, n (%)**	2 (25)	4 (50)	0.61
**LGE, % of MM**	5.3 ± 5.9	5.7 ± 4.6	0.91
**RVEDVi, mL/m**^**2**^	50 ± 7	48 ± 10	0.65
**RVESVi, mL/m**^**2**^	20 ± 6	18 ± 5	0.60
**RVSVi, mL/m**^**2**^	30 ± 8	30 ± 8	0.94
**RVEF, %**	59 ± 11	62 ± 7	0.65

*BNP*, B-type natriuretic peptide; *TNI*, troponin I; *LV*, left ventricle; *RV*, right ventricle; *i*, indexed; *EDV,* enddiastolic volume; *ESV*, endsystolic volume; *SV*, stroke volume; *MM*, myocardial mass; *LGE*, late gadolinium enhancement; Data expressed as mean ± 1 standard deviation.

Patients subsequently underwent surgical repair with establishment of a two coronary circulation that was achieved by direct coronary re-implantation in six and by the Takeuchi method in two patients. Additional creation of a central pulmonary artery banding was necessary in the patient with ventricular septal defect. Mitral valve repair was not performed in any patient. Mean aortic clamp time was 69 ± 9 minutes and mean bypass time was 124 ± 23 minutes. Mean duration of mechanical ventilation was 139 ± 174 hours and mean time on intensive care unit was 14 ± 10 days. Inotropic support was needed in all patients for a mean duration of 14 ± 10 days. Extracorporeal membrane oxygenation therapy (ECMO) was needed postoperatively in two patients (duration of 5 and 12 days) due to poor left ventricular function with impaired cardiac output after weaning from cardiopulmonary bypass. None of these two patients had myocardial scarring on CMR but these two patients had the most compromised LV function preoperatively (LV-EF of 10% and 11%). Patients with myocardial scars did not require ECMO support and did not receive longer ventilation therapy, inotropic drug support or ICU stay compared to patients without scar.

**Figure 3:**
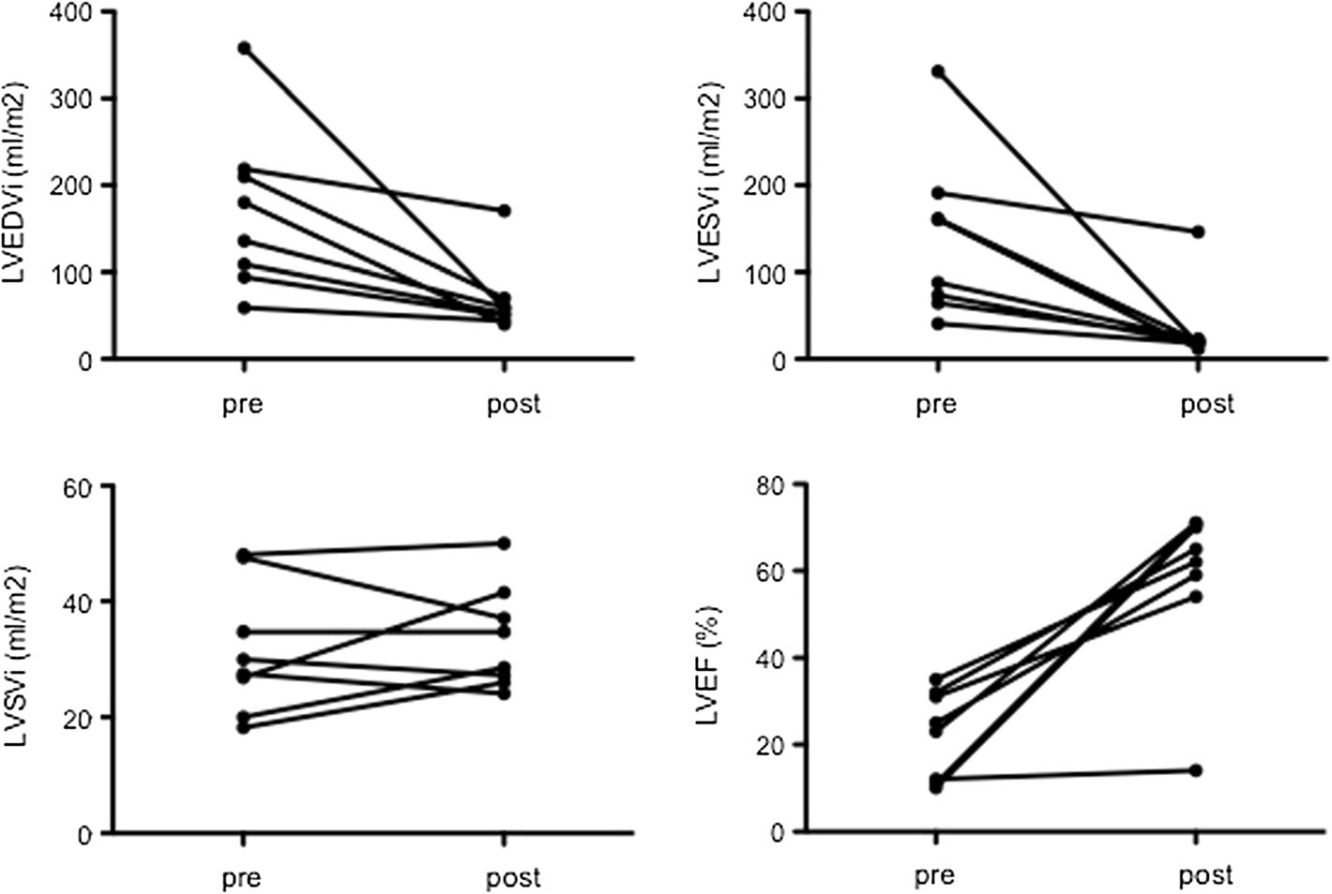
**Changes in left ventricular dimensions (enddiastolic, *EDV *and endsystolic volume, *ESV*) and function (stroke volume, *SV *and ejection fraction, *EF*) after coronary reimplantation surgery.** Note the lack of early functional recovery in one patient in whom apical myocardial scarring was detected.

At early follow-up CMR (mean 4.9 ± 2.5 months post-repair), LV size and function improved significantly in 7 of the 8 patients with mean indexed LVEDV decreasing to 68 ± 42 mL/m^2^ (p = 0.02) and a rise in mean LV-EF to 58 ± 19% (p < 0.001) (Figure 3). Moycardial mass (indexed to body surface area) decreased significantly from 91 ± 48 to 61 ± 30 g/m^2^ (p = 0.04). RV dimensions and function did not change significantly (Table 2).

In the patient with transmural antero- and inferolateral scarring prior to surgery, LV scar increased from 9.3 to 11.4% of LV mass but ventricular dimensions and function (EF at follow-up 62%) normalised post-surgery. The other patient with scarring before reimplantation was the only patient who showed almost no recovery of myocardial volume and function at early follow-up post-repair although scarring decreased from 1 to 0.6% of LV mass (Figure 2). However, at late CMR follow-up 1.5 years after surgery, LV function of this patient had improved markedly to an EF of 55%.

**Figure 4:**
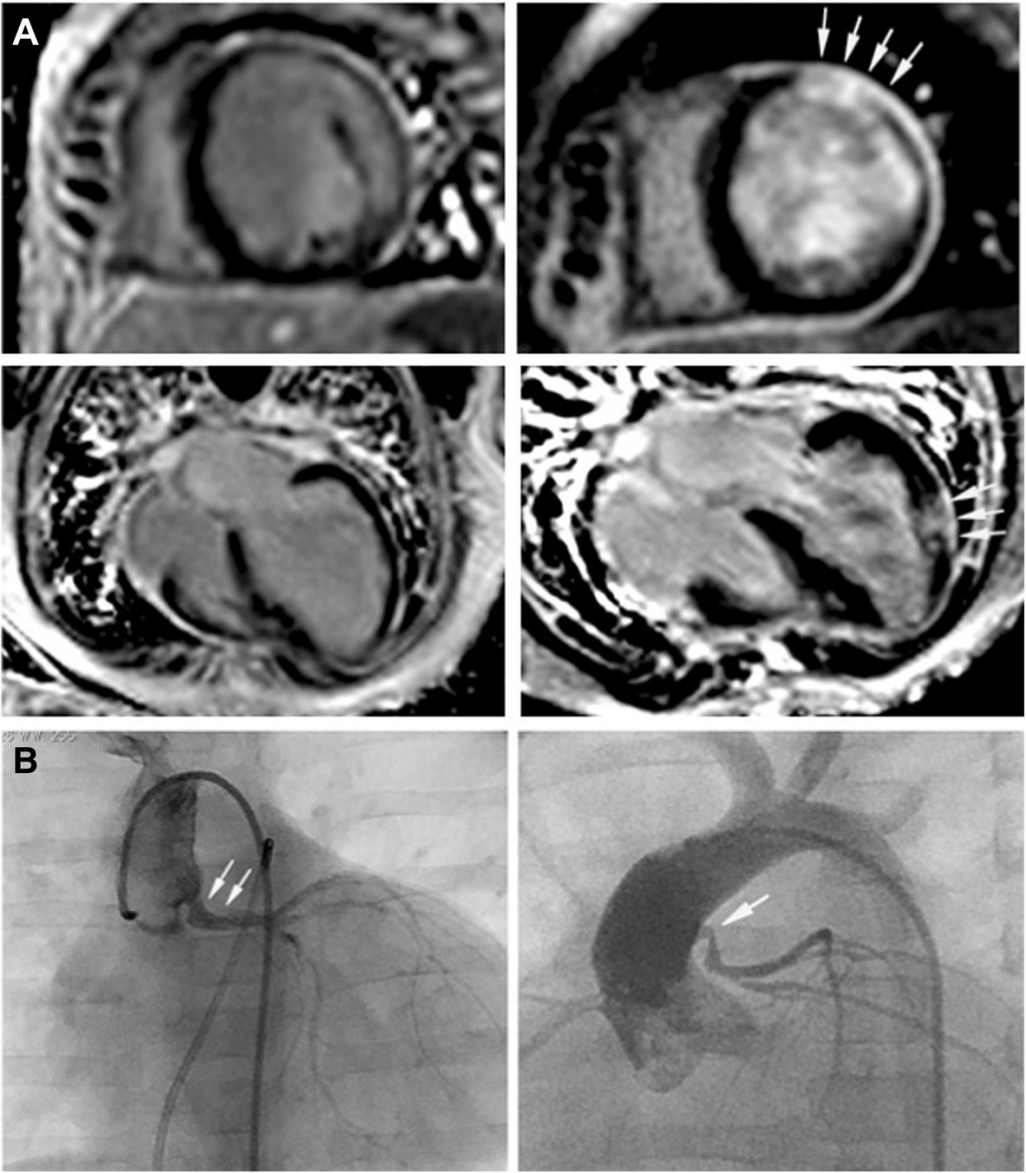
**New onset myocardial scarring in two patients post-repair. A** LGE images of the two patients who had no evidence of myocardial scarring pre-repair (*left*), but in whom transmural myocardial infarction (*arrows*) was detected postoperatively (*right)*. Despite this ischemic insult, recovery of LV dimension and function was sufficient in both cases. **B** Both patients subsequently underwent selective coronary angiography that revealed unobstructed coronary arteries in one (*left, corresponding CMR images at the top)*, but stenosis at the anastomosis (*arrows*) site in the other patient (*right, corresponding CMR images at the bottom*) who subsequently had to undergo re-surgery that confirmed the angiographic findings.

In two patients newly developed anterolateral and inferolateral transmural scarring at the midventricular level not evident before re-implantation was found at follow-up CMR although recovery of LV dimension and function were absolutely sufficient (Figure 4A). Both patients subsequently underwent cardiac catheterization showing an unobstructed origin of the re-anastomosed left coronary artery (LCA) in one patient while in the other patient coronary angiography revealed stenosis at the proximal level of the LCA (Figure 4B) necessitating re-surgery in this patient. Intraoperatively, LCA stenosis could be confirmed and was relieved by pericardial patch augmentation and re-anastomosis.

Levels of BNP and cardiac TNI (available in 7 of the 8 patients) decreased significantly from 1349 ± 765 to 90 ± 60 pg/mL (BNP) and from 0.34 ± 0.26 to 0.02 ± 0.01 μg/L (TNI) post-surgery (p < 0.001 and p = 0.02).

## Discussion

This study is the first to assess myocardial function and viability using cardiovascular CMR before and in the short-term follow-up in children after ALCAPA repair. We were able to show that despite diminished myocardial perfusion and consecutively often severely compromised LV function, myocardial scarring was preoperatively only present in the minority of our patients. Interestingly, improvement of myocardial function was independent of preoperative and new-onset myocardial scarring. Furthermore, pre- and post-repair CMR assessment can provide important clinical information as our results demonstrate this imaging modality may help to identify patients with coronary artery obstruction after coronary reimplantation.

In infants with ALCAPA, left-to-right shunting occurs when pulmonary vascular resistance gradually decreases causing a “coronary steal” that results in myocardial hypoperfusion. Consequently, ischemic dilated cardiomyopathy develops that is typically characterized by an enlarged and globally hypocontractile left ventricle. Histological studies of biopsy specimens taken from hypoperfused myocardium in ALCAPA patients revealed a variable degree of necrosis but potentially revivable myocardium [17,18]. Their hypothesis was that the ischemic but yet viable myocardium, also called hibernating myocardium, allows for quick myocardial recovery after coronary revascularization. In our study population myocardial scarring was only present in two of the eight patients, which confirms the idea that most of the ischemic LV myocardium is viable in ALCAPA-patients. Other myocardial viability studies using different imaging modalities were applied in the assessment of hibernating myocardium before surgical repair of ALCAPA. Finley and colleagues assessed myocardial perfusion using thallium-201 imaging and described anterolateral myocardial perfusion defects pre-repair that decreased after successful corrective surgery [8]. Yang and colleagues recently used myocardial perfusion/F-18 fluorodeoxyglucose imaging and demonstrated that the degree of myocardial viability was related to the clinical manifestations as well as cardiac function and that this imaging modality may predict functional recovery after repair [19]. Using LGE-CMR in two infants with ALCAPA that presented with lack of LV functional recovery early after repair, Browne and colleagues [16] detected distinct areas of LGE and concluded that the findings were the consequence of chronic ischemia resulting in fibrosis and they presumed that myocardial scarring probably preceded the reimplantation procedures. In this report, both patients subsequently underwent heart transplantation and the authors concluded that LGE-CMR might be useful in identifying those patients in whom coronary artery revascularization will fail and who should therefore primarily be considered for heart transplantation. In our study population, none of the LGE positive patients before ALCAPA-operation required ECMO-therapy and the immediate postoperative course showed no relation with myocardial scarring. The two patients in our group that had a complicated postoperative course including mechanical circulatory support showed the most impaired LV function prior to surgery, a known risk factor for postoperative survival [3,9,20]. Based on these results, assessment of myocardial scarring pre-repair will probably not influence surgical strategy in the majority of ALCAPA patients, although the indication and prognostic relevance of CMR-LGE imaging has to be determined in further studies including a larger number of patients.

There remains uncertainty about the potential factors that finally cause myocardial necrosis in some patients while in other patients ischemic myocardium is only at risk but will not infarct. Likely, the development of sufficient intercoronary collateralization maintains myocardial oxygen delivery thereby attenuating a total ischemic insult to the myocardium [21-24]. This idea is further supported by the study of Schwarz and colleagues who revealed only little extent of hibernating myocardium in the presence of large areas of infarction in hypoperfused LV myocardium in adult pigs, an animal study that clearly misses intercoronary collateral flow as an important factor [11,21,25]. Given the classic concept of “adult-type” of ALCAPA [26,27], intercoronary collateralization allows these patients to reach adulthood and protects the myocardium from irreversible damage, which, in turn, raises the question whether surgical repair is required in adulthood. Indeed, Komocsi and colleagues reported three adult cases with uncorrected ALCAPA and found only moderate ischemia and limited necrosis in two of the three patients that had normal or only mildly impaired LV function [28].

However, although our findings support the suggestion that only minor cell death occurs in infants with ALCAPA, troponin levels were elevated in six of seven patients at initial presentation demonstrating that chronic hypoperfusion, nevertheless, results some degree of ischemic insult to the left heart.

Despite markedly depressed ventricular function prior to surgery, most ALCAPA-patients show quick improvement of LV performance after repair with total functional recovery within 1 year after surgery [29-31]. Our study confirmed these findings as LV function normalized in 7 of the 8 patients in a short-term follow-up after repair. However, one of the patients in our study, in whom myocardial scarring was found preoperatively, showed delayed recovery at early follow-up CMR while the other LGE positive patient recovered quickly. The patient with absent early improvement in LV function showed delayed but almost full functional recovery within 18 months after surgical correction, a phenomenon that is in accordance with previous studies that found normalization of LV function can last up to 22 months [31]. Interestingly, this patient underwent surgical correction at the age of 1 year and was therefore the oldest patient in our study cohort. In the study of Jin and colleagues [7], patients older than 10 months of age showed less depressed LV function preoperatively, but more rapid regression of LV enlargement and complete recovery of LV function was observed in the younger patients which is probably related to the reduced duration of myocardial hypoperfusion with subsequent less ischemic myocardial insult.

Two patients in our group had new-onset scarring at the follow-up CMR although functional recovery was absolutely adequate. While coronary obstruction at the anastomosis site was found in one patient, coronary anastomosis was patent in the other patient. Interpretation of these findings is challenging because in the presence of relevant coronary stenosis complete recovery of myocardial function seems unlikely. Furthermore, we can only speculate about the reasons for the newly acquired scar despite an unremarkable surgical result in this patient. It remains unknown whether infarction occurred during surgical correction at cardioplegic arrest that represents an additional ischemic injury to the already hypoxic underperfused myocardium or if embolic infarction has occurred during or after surgery. The patient with patent coronary anastomosis was the one with the longest cross-clamp time (81 minutes), a finding that supports the hypothesis of intraoperative ischemic insult finally causing myocardial infarction.

Despite excellent surgical results after surgery with little or no mortality in most series and preserved ventricular function late after repair the long-term prognosis of patients after reimplantation of ALCAPA is unclear. Even after successful coronary reimplantation with subsequent recovery of LV function, the possibility of persistent or recurrent myocardial damage and subclinical ischemia is still present as recent CMR-studies using stress-perfusion and LGE imaging techniques revealed myocardial scarring [10,21,32]. In the study by Secinaro et al. [32] six patients presenting with typical signs of myocardial ischemia underwent LGE-CMR that showed subendocardial scarring in five of the six patients although left ventricular function and volume was normal. In another study by Fratz et al. [21], myocardial scarring in very little extent ranging from 0 to 11% of total myocardial mass was found in 71% of the patients after ALCAPA-repair but was not related to LV dimension, function or exercise capacity. No specific pattern of scarring could be determined as transmural and multifocal scarring were detected. The variable patterns of myocardial LGE observed in these and our study likely reflect different causes at various time points of myocardial injury that might have different prognostic implications. These findings illustrate the need for appropriate imaging before and after repair of ALCAPA and highlight the usefulness of LGE-CMR even in very young patients that may be helpful to assess hibernating myocardium that regains function after successful revascularization. In addition, further studies are needed to elucidate the endogenous repair mechanisms that are responsible for the re-remodeling of the LV in patients with ALCAPA.

### Study limitations

Our study is limited by the small patient size, but based on our results, we advocate further research in the pre- and postoperative CMR evaluation of patients with ALCAPA with the hopeful objective to establish criteria that include the impact of pre-existing myocardial scars on early postoperative course (thereby identifying patients who will require cardiac transplantation), overall recovery of ventricular function and long-term prognosis.

Two-dimensional CMR flow measurements in the pulmonary artery and the ascending aorta can reliably quantify left-to-right-shunt but were not part of our CMR studies. Therefore, we were unable to relate the degree of coronary steal to the presence of myocardial scarring.

Scar imaging using LGE-CMR is currently considered as the gold standard of assessing myocardial viability. However, besides metabolic imaging and assessment of contractile function, it represents only another imaging strategy to assess hibernating myocardium and even in adult patients with ischemic cardiomyopathy, there does not exist a well defined test that is suited to predict functional recovery of hibernating myocardium after revascularization procedures. The described patterns of diffuse fibrosis and necrosis in the study by Shivalkar and colleagues are probably missed by the used LGE-technique in our study which only detects regional or local scars. New CMR techniques such as contrast enhanced T1 mapping sequences may be able to quantify diffuse fibrosis. Furthermore, as the long-term effects of gadolinium in pediatric patients <2 years of age is yet unclear, we advice to use it with caution in these young patients [14].

## Conclusions

Despite diminished myocardial perfusion and compromised LV function, myocardial scarring was preoperatively only present in the minority of our patients. According to our experience, improvement of myocardial function was independent of new-onset myocardial scarring while the impact of preoperative myocardial scars still needs to be defined. Further studies are needed to elucidate the endogenous repair mechanisms that are responsible for the re-remodeling of the LV in patients with ALCAPA.

## Abbreviations

ALCAPA: Anomalous left coronary artery from the pulmonary artery; BNP: B-type natriuretic peptide; CMR: Cardiovascular magnetic resonance; ECMO: Extracorporeal membrane oxygenation; EDV/ESV/SV: Enddiastolic/endsystolic/stroke volume; EF: Ejection fraction; LCA: Left coronary artery; LGE: Late gadolinium enhancement; LV: Left ventricle; ICU: Intensive care unit.

## Competing interests

The authors declare that they have no competing interests.

## Authors’ contributions

HL, HA, DS and CA have contributed to the conception and design of the study. HL, KG and SR have contributed to the acquisition of data, analysis and interpretation of the data. MM, GK, CJ, JB, DS and CA have contributed to the drafting of the article and have revised it critically for important intellectual content. All authors read and approved the final manuscript.
